# Sugar Transport and Signaling in Shoot Branching

**DOI:** 10.3390/ijms252313214

**Published:** 2024-12-09

**Authors:** Joan Doidy, Yuhui Wang, Léo Gouaille, Ingrid Goma-Louamba, Zhengrong Jiang, Nathalie Pourtau, José Le Gourrierec, Soulaiman Sakr

**Affiliations:** 1 EBI Ecologie et Biologie des Interactions, Université de Poitiers, UMR CNRS 7267, 86073 Poitiers, France; joan.doidy@univ-poitiers.fr (J.D.);; 2 Institut Agro, Univ Angers, INRAE, IRHS, SFR QuaSaV, 49000 Angers, Franceleo.gouaille@inrae.fr (L.G.); jzr@njau.edu.cn (Z.J.); jose.gentilhomme@univ-angers.fr (J.L.G.); 3 College of Agronomy, Nanjing Agricultural University, Nanjing 210095, China

**Keywords:** axillary bud, shoot branching, sugar transport and signaling, apoplasmic and symplasmic unloading pathways, SUTs, SWEETs

## Abstract

The source–sink relationship is critical for proper plant growth and development, particularly for vegetative axillary buds, whose activity shapes the branching pattern and ultimately the plant architecture. Once formed from axillary meristems, axillary buds remain dormant or become active to grow into new branches. This transition is notably driven by the regulation of the bud sink strength, which is reflected in the ability to unload, metabolize and store photoassimilates. Plants have so far developed two main mechanisms for unloading sugars (sucrose) towards sink organs, a symplasmic pathway and an apoplasmic pathway, but so far limited investigations have been reported about the modes of sugar uptake during the transition from the dormant to the active outgrowth state of the bud. The available data indicate that the switch from dormant bud to active outgrowing state, requires sugar and is shortly preceded by an increase in bud metabolic activity and a remobilization of the stem starch reserves in favor of growing buds. This activation of the bud sink strength is accompanied by an up-regulation of the main markers of apoplasmic unloading, such as sugar transporters (sucrose transporters—SUTs; sugar will eventually be exported transporters—SWEETs), sucrose hydrolyzing enzymes (cell wall invertase—CWINV) and sugar metabolic pathways (glycolysis/tricarboxylic cycle—TCA; oxidative pentose phosphate pathway—OPPP). As these results are limited to a few species, they are not sufficient to provide a complete and accurate picture of the mode(s) of sugar unloading toward axillary buds and deserve to be complemented by additional studies in a wide variety of plants using systems integration, combining genetic, molecular and immunolocalization approaches. Altogether, we discuss here how sugar is a systemic regulator of shoot branching, acting both as an energy-rich molecule and a signaling entity in the establishment of the bud sink strength.

## 1. Sugar Transport from Source to Sinks

### 1.1. Long-Distance Transport of Sugars from Source to Sink Organs

Sugar transport in plants is a major determinant of growth, yield and stress resilience. It relies on the efficient and controlled allocation of sugars across plant organs through the phloem. This process is known as source-to-sink transport [1]. In this process, sugars mainly in the form of sucrose are synthesized in source organs (mature leaves) and translocated through the phloem to sink organs (such as roots, seeds or buds) where they are utilized for growth, storage and metabolism.

The first step in carbon source-to-sink transport consists of phloem loading where sugars are loaded into the sieve element-companion cell complex. In most species, sucrose is the main form of sugars for long-distance transport [2], but other plants can translocate other carbohydrates, such as polyols and raffinose [3]. Phloem loading can be either apoplasmic, involving sucrose transporters like SUT (sucrose transporter) and SWEET (sugar will eventually be exported transporter), or symplasmic through plasmodesmata depending on the species (for a review, see [4]). Then, sucrose and other sap solutes are translocated through the phloem vessels following the pressure flow mechanism. In the transport phloem, sucrose can be unloaded and retrieved to supply flanking tissues [5]. In the last step of long-distance transport, sucrose is unloaded to supply sink organs [6].

### 1.2. A Spatio-Temporal Control of Sugar-Unloading Pathways

**Figure 1:**
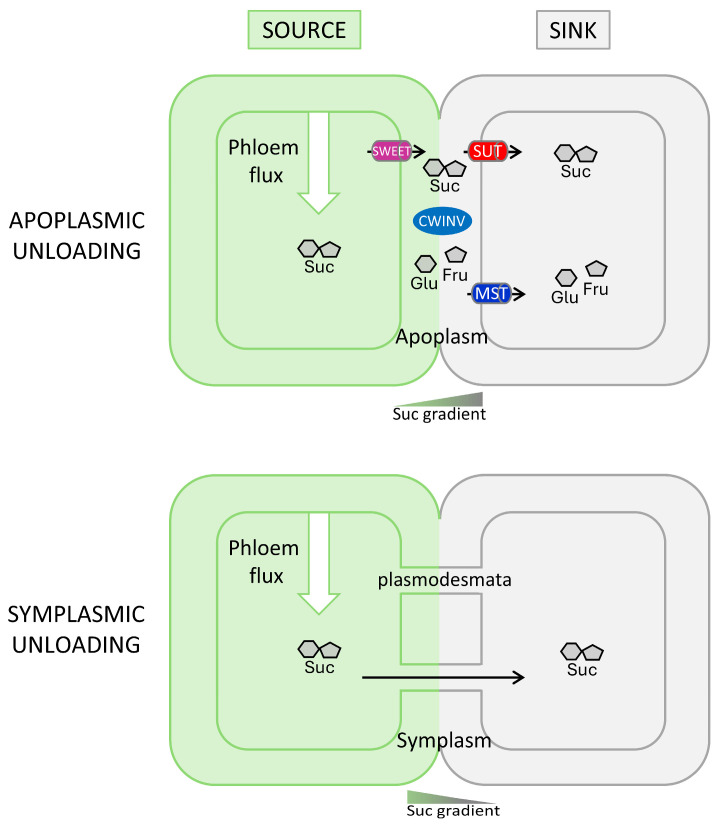
Schematic representation of the apoplasmic and symplasmic unloading pathways. In apoplasmic connected sinks, sucrose (suc) is unloaded actively by sugar transporter proteins against the concentration gradient. Apoplasmic unloading is often associated with the activation of sucrose transporters (SUTs), monosaccharide transporters (MSTs) and sugar will eventually be exported transporters (SWEETs), as well as cell wall invertase (CWINV) enzymes maintaining a high sink strength and producing energy-rich glucose (glu) and fructose (fru) molecules required for the metabolic activity of growing organs (top panel). Symplasmic phloem unloading is driven by the sucrose gradient and a high xylem pressure flow, following a symplasmic route through a network of open plasmodesmata connections (bottom panel). For simplification purposes, the representation of intermediate cells, such as mesophyll cells in source leaf, companion cells and sieve elements in the transport phloem and parenchyma cells in sink tissues are not represented. Please refer to these reviews describing intermediate spatial barriers of specific sinks, such as root, seed and fruit organs [8,14,19,20]. In this figure, both the apoplasmic and symplasmic pathways are represented in separate panels for illustrative purposes; however, both mechanisms could concur in sink organs. For instance, apoplasmic unloading (via SUT and SWEET) could contribute to a lesser extent in symplasmically connected sinks. In contrast, apoplasmic unloading can be associated with the deposition of callose in plasmodesmatal connections, thus restricting the symplasmic route.

In contrast to the loading process, sugar unloading can occur both via the apoplasm or through the symplasm in the same individuals (Figure 1). Indeed, unloading pathways rather depend on the type of sink organs [7]. In seed organs, sugar transfer from the apoplasmic separation between maternal and filial tissues involves facilitated and active transport systems [8,9]. In various grain species, such as barley, fava bean and pea seeds, sugar transfer toward the embryo and the endosperm from maternal tissues involves SWEET and SUT membrane transporters, as well as sucrose-cleaving enzymes such as invertase (INV), maintaining a high sink strength for efficient apoplasmic unloading [10,11,12]. In contrast, in root organs specialized in storing sugars, sucrose is transferred to storage parenchyma cells via the symplasmic pathway and then accumulated in vacuoles [6,13]. However, beyond the root tip, no further symplasmic unloading is observed from the metaphloem suggesting an apoplasmic unloading mechanism [14]. Transport of sugar via the apoplasmic pathway may involve active SUTs [14,15,16] and SWEET facilitators [17,18], even if the high sucrose concentration in the sieve tube does not require active transport. 

Also, a temporal switch from one unloading pathway to another can occur during sink development. In potato, for instance, during tuber development, sucrose phloem unloading is first apoplasmic, and then becomes symplasmic [21,22]. Similarly, in some fruits, such as tomato or grape berry, both unloading pathways occur at different stages of fruit development. Symplasmic unloading occurs at the early stage of fruit development when the sugar level is low and then shifts to apoplasmic during ripening as soluble sugars start to accumulate [21,22,23]. 

## 2. Axillary Bud as a Major Carbon Sink

### 2.1. Axillary Bud, a Critical Carbon Sink for Plant Branching and Organismal Development

Sink strength refers to the capacity of a sink organ to import and accumulate resources, such as carbon photoassimilates. It reflects the ability to lower the concentration of photoassimilates in the sieve tube cells at the sink, thereby creating a hydrostatic pressure gradient, facilitating long-distance transport between source and sink [24]. A plant can thus be viewed as a complex network of sources and sinks, organized hierarchically according to the type of sink organ and its developmental stage [1]. The demand for sugars in the sink organ (sink strength) determines the allocation of carbon from the phloem to competing sink organs. Therefore, the growth rates of sinks can be seen as a good reflection of the sink strength [25,26]. Various strategies are implemented to decrease sucrose concentration depending on the type of sink (utilization vs. storage). In growing sinks, sucrose is utilized for metabolism in large quantities, suggesting a high supply of sucrose, implying that this flux is facilitated through a symplasmic unloading pathway [27]. Spatial and temporal regulation of the carbon sink strength also occurs throughout plant development. For instance, roots, immature leaves (before their source transition) and buds are major sinks attracting a substantial proportion of source photosynthates during the early stages of plant development, whereas fruits and seeds become important sinks during the reproductive phase [24].

The plant shoot architecture is considered as an important crop yield trait, mostly determined by the number of shoot branches that result from vegetative axillary buds [28]. The plasticity of shoot architecture contributes to stress resilience and the aesthetic quality of ornamental plants [29,30,31,32]. Furthermore, shoot branching is a process finely regulated by a wide array of factors (listed in Table 1), which raises the question of how shoot branching may evolve under future growing conditions, where plants will be exposed to a variety of changing climatic factors. Given that extensive research has focused on the hormonal regulation of shoot branching, as well as that the axillary bud is a critical sink organ for the development of most aboveground plant organs, we conclude that there is a need for further research into the molecular mechanism governing the sink strength of the outgrowing bud to sugars. Indeed, the capacity of the bud to import, accumulate and utilize carbon resources is crucial for determining its ability to develop into a branch and shape the shoot architecture. In contrast to other sink organs such as fruits, seeds and lateral roots, the sink-driven processes of bud outgrowth are still poorly understood, and this lack of knowledge is detrimental to the future improvement of plant architecture in the context of changing climate and reducing inputs. 

This review aims to summarize our current knowledge on the diversity of the mechanisms contributing to the sink strength of axillary buds, thus highlighting the main questions that need to be addressed in the near future. After a brief introduction to the axillary bud structure, we will discuss the major changes in sugar metabolism and transport induced shortly before the onset of bud outgrowth. We will then review the different sugar signaling pathways identified so far in buds and how sugar may contribute to bud sink strength in interaction with cytokinin (CK). Numerous avenues of research will be proposed for future investigations, as having a better understanding of bud sink strength would provide timely applications in crop improvement and ornamental plant production.

### 2.2. Structure, Establishment, and Activity of Axillary Vegetative Bud

The axillary vegetative bud originates from a group of meristematic cells in the stem of a parent shoot just above the insertion point of a leaf primordium [28,66]. In brief, axillary bud development involves three distinct steps: (i) axillary initiation corresponds to the maintenance of a meristematic cell population under auxin action through the repression of the *SHOOT MERISTEMLESS* (*STM*) transcription factor; (ii) axillary activation involves a high level of STM expression due to the specific expression of the *REVOLUTA* (*REV*) and *LATERAL SUPPRESSOR* (*LAS*) genes in the leaf primordia and the expression of a complex molecular regulatory network including *CUP-SHAPED COTYLEDON 2* (*CUC2*), a transcription factor of the NAC family, and *REGULATOR OF AXILLARY MERISTEM1* (*RAX1*), an R2R3 MYB-like transcription factor; (iii) the initiation or emergence phase (formation of meristematic dome) is characterized by the *de novo* activation of *WUSCHEL* (*WUS*) by cytokinin (Arabidopsis Regulator Response 1: ARR1) and the establishment of the WUS-CLAVATA3 (CLV3) feedback loop (for review, see [67,68]). The position of primordia is defined by auxin and cytokinin distribution (for review, see [32]). High levels of auxin trigger new lateral organ initiation [69,70,71], whereas cytokinin creates an inhibitory field that prevents the formation of new lateral organs [72,73]. Sugars (i.e., sucrose and glucose) have long been known to act as mitogenic regulators by upregulating cyclin-dependent kinases (*CDK*) and cyclin (*CYC*) expression, critical for G1 to S transition (for review, see [74]); *TPR-DOMAIN SUPPRESSOR OF STIMPY* is required for G2 to M transition [75]; and *SHOOT MERISTEMLESS* (*STM*) [76] is a transcription factor TF essential for the establishment of the meristem and its maintenance [77]. STM suppresses differentiation and promotes cell division by inducing the expression of *CYCLIN D3* (*CYCD3*) and *ISOPENTENYL TRANSFERASE7* (*IPT7*), which encodes a key enzyme involved in cytokinin (CK) biosynthesis [78,79]. Sugars also increase auxin accumulation in meristem [80] by stimulating the biosynthesis of the main three auxin biosynthesis pathways (Indole-3-acetamide (IAM), indole-3-acetaldoxine (IAOX) and Indole-3-acetonitrile) [81]. Bud formation therefore requires sugar not only as a carbon and energy source to meet the high metabolic demands associated with mitotic and organogenesis activity but also as signaling molecules to coordinate this process according to sugar availability. These results indicate a high sink for sugar in the bud under formation, although the mechanism of sugar supply is still unknown.

The formation of axillary buds in each leaf axil is genetically controlled but not influenced by endogenous and environmental factors. Meristematic tissues are shared within three cellular zones: (i) a central zone (ZC), located at the top of the meristem, containing slowly dividing stem cells; (ii) the peripheral zone (ZP), forming a ring around the central zone, with frequently dividing cells that differentiate to form new organs; and (iii) the “rib zone” or medullary zone (ZM), located below the central zone and composed of cells producing the internal parts of the stem [82]. Subsequent growth of this meristematic area leads to fully developed axillary buds consisting of a miniature shoot—meristematic zone and pre-formed leaf primordia- enclosed by scales and attached at its base to the parent stem. The formation of axillary buds in each leaf axil is genetically controlled but not influenced by endogenous and environmental factors. Once the bud is formed, it can either directly produce new branches, in the case of sylleptic buds, or enter a dormant phase, as with proleptic bud [83]. Proleptic buds may remain dormant or be activated to grow into new shoots. Three types of dormancies have been identified, including para-dormancy, which is mainly due to the inhibition of axillary bud outgrowth by the rapidly growing stem tip, referred to as apical dominance [84]. The release of apical dominance (e.g., decapitation) results in the development of axillary buds into new branches, whose number and spatio-temporal arrangement contribute to the final shoot architecture of the plant.

## 3. Sugar Supply Towards Axillary Bud

### 3.1. Sugar Supply During the Transition from Dormant to Active Buds

The axillary bud is a true sink organ, unable to produce the organic molecules (photoassimilates) necessary for its own growth and development. Numerous studies reported a link between dormancy and a low sugar level in axillary buds, pointing out the weak sink activity to compete for the photoassimilates produced by the parent plant [85]. Tarancón et al. (2017) [86] highlighted that dormant buds in annual and woody species shared an accumulation of carbon starvation-like transcripts, that might result, at least in part, from an epigenetic regulation involving the binding and phosphorylating activity of Sucrose non fermenting related Kinase 1 (SnrK1), a key regulator in adjusting cellular metabolism during starvation, stress conditions, and growth-promoting conditions ([87]; For review see [88]). By contrast, the onset of bud outgrowth in several species including *Quercus robur* [89], *Prunus persica* [90,91], and *Rosa hybrida* [92], was preceded by a stimulation of bud sink strength, and remobilization of stem-stored sugars for the benefit of growing buds [92,93,94,95,96]. In wheat, dormant buds exhibited a significantly lower level of sugar compared to growing ones, associated with the upregulation of *DIN6* (*dark inducible 6*), a gene marker of sugar starvation [97]. In sorghum, reducing the photosynthetic leaf area of plants through defoliation inhibited bud outgrowth [98] and the dormant buds of *Phytochrome B* (*phyB*) mutants showed a high level of *DIN6* [99]. A causal relationship between sugar availability and bud outgrowth was also uncovered in pea, where the exogenous sugar supply via phloem sap was sufficient to induce bud outgrowth even in intact plants subjected to apical dominance [100]. Finally, the use of feeding experiments with explant stems (a bud-bearing node), initially established in Arabidopsis [101], was proven useful for understanding the growing bud’s need for sugar, and later to desiccate the interaction between sugar and hormone signaling in the regulation of bud outgrowth. These feeding experiments showed that in contrast to non-metabolizable sugars (e.g., sorbitol, mannitol), metabolizable sugars (sucrose, glucose and fructose) induced outgrowth in a dose-dependent manner in different plant species [80,102,103,104,105,106,107,108]. These outcomes clearly indicate that sugars potentially stored in the stem or sugars derived for stem photosynthesis are not sufficient to promote bud outgrowth [80], hence; sugar supply from the parent shoot is critical for bud outgrowth, raising the question of the metabolic activity and the mode (apoplasmic/symplasmic) of sugar supply towards growing buds.

### 3.2. Sugar Metabolism Activation in Growing Axillary Buds

The activity of sugar metabolism is known to be central to the establishment of the sink strength of multiple non-photosynthetic organs [22]. This was also the case for axillary buds, where the transition of the bud from the dormant to the active state was strongly associated with the reprogramming and stimulation of sugar metabolism in different species. For instance, invertase (INV) enzymes hydrolyze sucrose unloaded from the phloem into energy-rich hexoses (glucose and fructose) and maintain a high carbon sink strength; thus, their induction is often associated with actively growing sinks [109]. In rose, a vacuolar invertase (*RhVI1*) was considered as a marker of bud outgrowth because its high expression and activity were among the earliest changes preceding this process [92,102]. In line with this, the defective mutants of vacuolar invertase (*VINV*) in potato showed a suppression of bud elongation [110]. Cell wall invertase (CWINV), another sucrose-degrading enzyme, was associated with bud growth activity in maize [111] and sorghum [99]. Transgenic Arabidopsis plants overexpressing a cyanobacterial fructose-1,6-bisphosphatase-II in the cytosol (AcF), a key enzyme in glycolysis that catalyzes the hydrolysis of D-fructose 1,6-bisphosphate to D-fructose 6-phosphate and inorganic phosphate, had elevated sucrose and hexose levels and an increased number of lateral shoots [112], further supporting this tight relationship between the activity of sugar metabolism and the ability of buds to grow out. More recently, using chemical and molecular approaches, Wang et al. (2021) [108] identified a key role for two major carbon metabolism pathways—the glycolysis/tricarboxylic acid cycle (TCA) and the oxidative pentose phosphate pathway (OPPP)—in bud outgrowth. Indeed, disruption of at least one of these two sugar pathways, by supplying the buds with inhibitors of sugar metabolism (2-deoxyglucose and 6-aminonicotinamide, inhibiting the glycolysis/TCA cycle and the OPPP, respectively), reduced bud growth activity in both the decapitated plants and the one node-cutting system [108]. These results indicate that the increase in sugar metabolism prior to the activation of bud growth could be part of the network mechanisms, establishing the carbon sink strength and allowing the bud to meet the high metabolic activity required for its transition from the dormant to the active stages.

### 3.3. A Possible Switch from Symplasmic to Apoplasmic Unloading Pathways During Axillary Bud Developmental Transition

**Figure 2:**
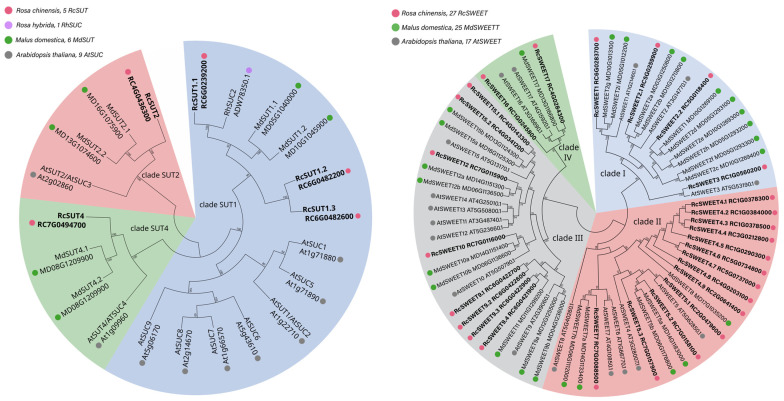
Phylogenetic trees of the SUT and SWEET families in rose and apple tree, two important models for shoot branching in horticulture and fruit agriculture. (Left panel): Sucrose transporters (SUTs) are major components of long-distance transport of sugars from source to sink organs [115]. (Right panel): Transporters of the SWEET (sugar will eventually be exported transporter) family facilitate sugar export from plant cells [116]; therefore, SWEETs represent key candidates involved in carbon partitioning in both source and sink organs [117]. Here, rose transporters (RcSUT and RcSWEET) were mined by BlastP in the *Rosa chinensis* genome [118], while *Malus domestica* (MdSUT and MdSWEET) was retrieved from two publications [119,120]. The sugar transporter accessions were named upon phylogenetic grouping with Arabidopsis SUT and SWEET families (AtSUT and AtSWEET).

The transition from a dormant axillary bud to active bud outgrowth implies the integration of complex environmental and internal factors. One key endogenous resource and signal is the supply of sugar from the parent shoot to the meristematic bud, which is critical to induce bud outgrowth. According to the nutritional hypothesis, the growing shoot tip acts as a dominant carbon sink, diverting sugars away from axillary buds and maintaining bud dormancy [113,114]. In pea, decapitation led to photoassimilate reallocation to the axillary buds before any earlier sign of bud outgrowth [100]. In rose, auxin supplied in the stem of one node-cutting system downregulates the expression of a sucrose transporter (*RhSUC2*—*Rosa hybrida sucrose transporter 2*; Figure 2) and thereby prevents buds from gaining access to sugar [103]. Consistent with this, the availability of sugar to axillary buds is an integral part of the activation mechanisms of bud outgrowth, and the unloading of sugar to the growing bud is a prerequisite step in the transition from bud formation to outgrowth.

**Figure 3:**
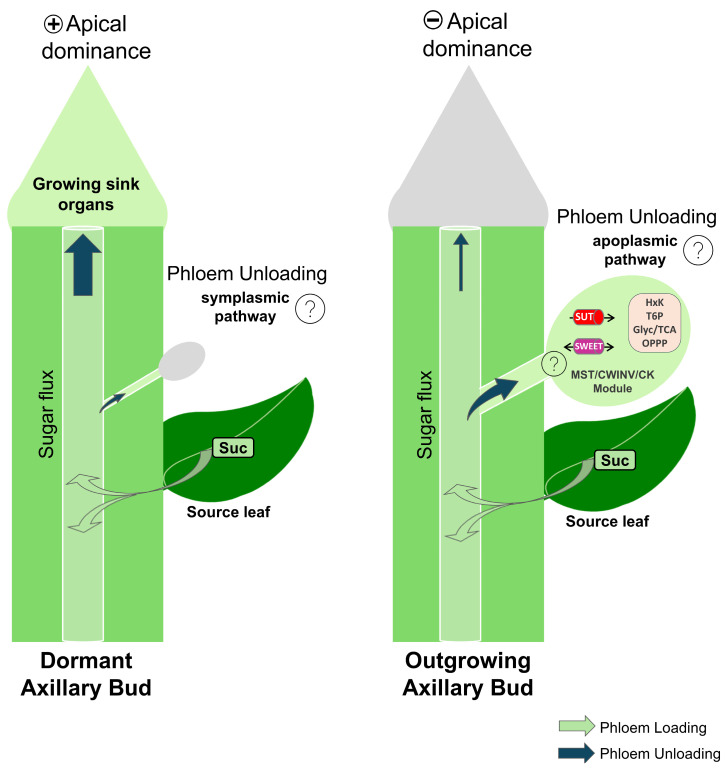
Schematic representation of the hypothesized mechanisms for phloem unloading during the transition from a dormant to an actively growing axillary bud and different sugar signaling pathways within the bud. In axillary dormant buds, marked by a low sink strength due to the diversion of sugar by the actively growing tip of the stem (e.g., apical dominance), sugar unloading is suggested to primarily operate through the symplasmic pathway via plasmodesmata. Upon transition to active outgrowth (e.g., loss of apical dominance), the bud sink strength is highly stimulated, as reflected by a significant stimulation of the metabolic activity, Glyc/TCA (glycolysis/tricarboxylic acid cycle), and OPPP (oxidative pentose phosphate pathway), sugar unloading shifts predominantly to the apoplasmic pathway, involving the following key transport proteins: SUT (sucrose transporters), SWEET (sugar will eventually be exported transporters), MSTs (monosaccharide transporters) and sucrose hydrolyzing CWINV (cell wall invertase) enzymes, considered to be the main markers of apoplasmic unloading. The MST/CWINV/CK (Cytokinin) module indicates that cytokinin can play a significant role in the sink strength of bud for sugar by upregulating CWINV and MST expression. The potential involvement of symplasmic unloading during bud outgrowth is not fully excluded. Sugar plays a signaling role, and three signaling pathways (represented in light blue rounded square) have been identified as involved in bud outgrowth: the T6P- (Trehalose 6P), HxK- (Hexokinase dependent pathway), and glycolysis/TCA-and OPPP-dependent pathways (Glyc/TCA and OPPP). Sugar loading from source leaves is indicated by green arrows with ascending and descending fluxes feeding shoot and root systems respectively. Carbon flux between the shoot tip and axillary buds is shown by blue arrows, with the thickness of the arrow representing the intensity of the sugar flux (competition for sugar). The question marks signifies that the precise spatiotemporal positioning of these transporters remains an open area for further investigation.

No prior research has clearly demonstrated the mechanism of sugar supply (sugar unloading) during the formation and the outgrowth of buds. According to Kebrom and Doust (2022) [85], two different mechanisms, the symplasmic and apoplasmic pathways (Figure 1), could be involved. They posited that bud formation relies on symplasmic sucrose supply and a transition to apoplasmic supply occurs during bud outgrowth (Figure 3). This shift from the symplasmic (through plasmodesmata) to the apoplasmic (via active transport system) unloading paths has been reported for certain sink organs and reflects to some extent a symplasmic isolation of the sink organ from the parental tissue. In developing seeds, the maternal seed coat and filial embryonic tissues are isolated by an apoplasmic barrier, thus sugar fluxes shift from symplasmic to apoplasmic in between seed compartments, as evidenced by the spatio-temporal regulation of genes encoding sugar transport systems [8,9]. In addition, during fruit maturation and ripening, a temporal shift from the symplasmic to the apoplasmic unloading pathways is accompanied by the coordination of sugar transporters and invertase enzymes as well as the deposition of callose at the plasmodesmatal cell walls [20]. Consistent with these findings, a possible switch from the symplasmic to the apoplasmic unloading pathways may also occur during the developmental transition from dormant to active buds based on the concurrent induction of genes encoding sugar transporters and invertases (see below). 

Thus, during axillary bud meristem initiation and bud formation, sugars would be supplied via the symplasmic pathway, as is the case for root tips and young leaves. An activation of the apoplasmic pathway would instead be associated with the transition from dormancy to the active stage, as is suggested in sorghum, sugarcane and maize (reviewed in [85]). In sorghum, Kebrom and Mullet (2016) [99] showed co-expression of *CWINV* and a *SWEET* gene, an orthologue of Arabidopsis *AtSWEET15*—involved in sucrose efflux from the maternal seed coat to the apoplasm for translocation to the embryo—during bud outgrowth [121]. In addition, SWEET11 and SWEET15 members also have a key role in seed filling in Arabidopsis, rice, and soybean [121,122,123]. In *Chrysanthemum morifolium*, the overexpression of the plasma membrane *CmSWEET17* resulted in an increased rate of bud outgrowth and more interestingly, CmSWEET17 could be involved in the process of sucrose-induced axillary bud outgrowth [124]. Interestingly, *CmSWEET17* overexpression lines showed a concurrent upregulation of auxin transporter genes, indicating that SWEET17 may be engaged in sugar-mediated axillary bud outgrowth via the auxin transport pathway [124,125]. In Arabidopsis, *SWEET11*, *SWEET12* and *SWEET13*, which are expressed in source leaves and involved in apoplasmic phloem loading, could regulate the supply of sucrose towards sink organs, including buds, while *SWEET13* and *SWEET14*, expressed in or near to axillary buds [126], could increase the local capacity to deliver sucrose to axillary buds [127]. In rose, among the four identified sucrose transporters, only the expression of *RhSUC2* (Figure 2) was positively correlated to bud’s ability to grow out in decapitated plants under light condition [103]. These authors showed that the expression of *RhSUC2* was restricted to the stem and the outgrowing bud, significantly induced as early as 48 h, before the onset of bud elongation at 96 h. Moreover, *RhSUC2* expression was repressed by exogenously applied auxin, the main systemic repressor of shoot branching [128,129]. The potential involvement of SWEET in *Rosa* bud activation (Figure 2) was also suggested by Roman et al., (2016) [130], who reported that cytokinin promoted bud outgrowth by upregulating the expression of *RhSWEET10*. Thus, SWEETs, as sugar transporters, facilitate the export of sugars (clade III members mainly transport sucrose, while clade II members transport monosaccharides) and play crucial roles in carbon supply for several sink organs [122,131,132]. Recently, three SWEET candidates were shown to be possibly involved in the accumulation of sugar during the floral bud transition from the undifferentiated stage to the physiological differentiation stage [133]. Here, we may also posit that CK signals promote bud outgrowth through the transcriptional activation of *INV*, *SUT* and *SWEET* genes [99,130], as these genes present a signature of “*cis*” regulatory elements related to phytohormonal responses in their promoter region. As a result, the spatio-temporal regulation pattern of *SUT*, *SWEET* and *MST* (sugar transport system), and *INV* (sucrose-hydrolyzing enzyme) could drive the carbon sink strength and the sugar fluxes to the buds, thus promoting bud outgrowth (Figure 3). In axillary buds, to the best of our knowledge, only two transporter genes have been studied so far in rose species (*RhSUC2* [103] and *RhSWEET10* [130]); several other candidates deserve further investigation (Figure 2). Such candidates may represent potential applications for agricultural and horticultural production.

However, the co-existence of symplasmic and apoplasmic pathways during bud outgrowth cannot be completely excluded as is the case for other sink organs (Figure 1) and further investigations are still required. The regulation of symplasmic unloading by changes in plasmodesmata has been reported in storage roots of cassava [134]. In Arabidopsis, Paterlini et al. (2021) [135] investigated whether an increase of callose deposits in the plasmodesmata of companion cells and parenchyma could disturb the ability of the axillary bud to grow. Callose deposition in plasmodesmata restricts symplasmic traffic of promoting growth factors such as hormones and nutrients, thus promoting the dormancy stage of buds [136]. Their results showed that high levels of callose accumulation did not alter bud outgrowth, whereas the subsequent growth rate of the activated buds was slightly inhibited. These results suggest that symplasmic unloading also contributes to sustained bud outgrowth, but it would be important to replicate such experiments in other species, including monocots and dicots, to accurately assess the respective roles of each of these two sugar-unloading pathways during plant branching process.

## 4. Sugar as a Signal Molecule with a Potential Role in the Establishment of Axillary Bud Sink Strength

Beyond its critical trophic role in bud outgrowth, a growing body of evidence from several species indicates that sugar also operates as a signal for this process. The results of feeding experiments based on the use of non-metabolizable sugar analogues of sucrose (e.g., palatinose, lactulose), as the sole carbon source for growing buds, revealed their ability to induce bud outgrowth in a manner similar to sucrose in *Rosa* [80,102] and in pea [106]. These findings paved the way for identifying three main sugar signaling pathways involved in shoot branching. Firstly, trehalose 6-phosphate (Tre6P)—a proxy for sucrose availability in plants [137]—increased very early after the decapitation of the shoot apex (within 6 h), together with the increase in bud size [106]. This Tre6P-dependent pathway positively regulates axillary bud outgrowth, either locally—transgenic lines with lower levels of Tre6P in buds displayed a strong delay in bud release—or in a systemic manner as Arabidopsis lines with an elevated level of Tre6P in the vasculature displayed more branches, more likely through enhanced sucrose allocation towards buds [135]. Secondly, the hexokinase pathway—considered the main glucose sensor in plants [138]—was demonstrated to be positively involved in shoot branching in Arabidopsis and *Rosa* [139]. The hexokinase loss-of-function mutant in Arabidopsis (*athxk1*) displayed a significant decrease in branch numbers [139]. This phenotype was associated with a high sensitivity of the buds to stem auxin and strigolactones, two major inhibitors of shoot branching, and a drop in their ability to synthesize and transduce cytokinin, an inducer of shoot branching, underlying that the hexokinase pathway could interact with phytohormones controlling shoot branching. Thirdly, Wang et al. (2021) [108] highlighted that sugar metabolism—glycolysis and the tricarboxylic acid cycle (TCA) and the oxidative pentose phosphate pathway (OPPP)—are central to the regulation of bud outgrowth in *Rosa*. More importantly, these two processes were antagonistically regulated by sugar availability and auxin, and the associated sugar signaling pathways directly modulated the transcriptional activity of the transcription factor *BRANCHED1* (*RhBRC1*), a master inhibitor of shoot branching [140]. Beside these three sugar signaling and metabolic pathways, Otori et al. (2019) [141] reported that AtSTP1, a monosaccharide transporter in *Arabidopsis thaliana*, acts as a factor for the regulation of shoot branching depending on the extracellular sugar contents. Taken together, these findings suggest that the bud has evolved multiple complementary mechanisms to sense sugar availability, which is critical for fine-tuning its outgrowth according to the carbon status of the plant. 

It has recently been demonstrated that sugar is a powerful systemic regulator of shoot branching, as it provides more carbon and energy for bud growth, but also triggers their release from dormancy and their development into a new shoot [100] by antagonizing the inhibitory effect of auxin in a concentration-dependent manner and reducing the sensitivity of bud to strigolactones, a repressor of bud outgrowth [80,142,143]. The role of sugar in the establishment of bud sink strength might be supported by its ability as a signaling entity to induce CK synthesis in the vicinity (node tissue) [80] and inside the bud [144]. CK has been widely reported as a key player of plant sink strength [145,146,147,148], including in axillary buds [99,110,130]. CK has been shown to trigger bud outgrowth through the promotion of cell division and sink strength [149]. However, even though CK is an inducer of bud outgrowth, CK alone fails to induce bud outgrowth in the absence of sugar (buds incubated on sugar-free medium or non-metabolizable sugar) in *Rosa* and in pea [80,144]. Sugars require CK to restore the decreased branching phenotype due to unfavorable light conditions (darkness and low light intensity) in *Rosa* [48,130]. These findings suggest that sugar and CK may act cooperatively in the establishment of sink strength, as is the case in other processes (for a review see [74]) and further studies are needed to clarify whether CK acts downstream of sugar. Consistent with this hypothesis, Dun et al. (2006) [150] reported that direct application of CK does not promote bud outgrowth, possibly due to the limited availability of sugar to meet the high demand for carbon and energy of the growing bud [85]. In addition, the promotive effect of CK, independent of sugar, on the bud outgrowth of detached stems [110] would indicate that detached buds from potato stem nodes and/or bud-bearing nodes intrinsically contain sufficient sugars for their growth in response to CK. These findings are very promising and should be further investigated in other species and extended to new questions, in particular to the relationship between sugar availability and CK synthesis and sensitivity for bud growth.

## 5. Conclusions and Perspectives

The carbon sink strength of axillary buds determines their ability to grow and break dormancy. Based on the literature available to date, two hypotheses for sugar unloading in buds are still under discussion: bud break would be accompanied by a shift from symplasmic to apoplasmic unloading, while, in addition to the symplasmic pathway, the apoplasmic pathway is triggered prior to bud break, resulting in the combined involvement of these two mechanisms (Figure 3). It is not ruled out that different unloading mechanisms are involved for sugar supply towards axillary buds in monocots and dicots. This situation reinforces the urgency of conducting further experiments to gain insight into these mechanisms. The first experiments would be based on the use of specific fluorescent probes of the symplasm (plasmodesmata) and apoplasm to determine their relative importance in both dormant and outgrowing buds [151]. Similarly, the identification and the functional characterization of sugar transporters involved in the apoplasmic pathway during bud break would be valuable for distinguishing these two pathways. Indeed, in seed development, for example, a symplasmic pathway facilitates the transport of sugars from maternal vascular tissues to seed coats, after which they are transferred to the apoplast via the apoplasmic pathway. Therefore, localizing sugar transporters within various bud structures and identifying their specific roles remain a key challenge. Moreover, the characterization of the branching phenotypes of different sugar transporter mutants will be a great help in functionally validating the exact role of each sugar transporter, paving the way to understanding their regulation by different endogenous (branching related hormones) and exogenous factors (e.g., light, water stress) and their localization within buds. These investigations are required for a better understanding of the role of sugar, as a systemic regulator of branching, in the triggering of the sink strength of buds, in interaction with the main branching-related hormones. 

## Figures and Tables

**Table 1:** The influence of different factors on shoot branching. This table summarizes the key factors known to significantly affect plant architecture through source/sink relationships and other mechanisms.

Factors	Processes	Species	References
Developmental stages	Apical dominance	Several species (Arabidopsis, pea, tomato, Petunia, rose, etc.)	For review, see [33]
	Flowering	Tomato and tobacco	[34]
		Rose and strawberry	[35,36]
	Fruits load	Arabidopsis	[37]
		Coffee	[38]
		Olive and Citrus	[39]
Light	Light quality	Petunia	[40]
		Rose	[41,42]
		Dallis and ryegrass	[43]
		Sorghum	[44,45]
		Arabidopsis	[46]
	Light intensity	Rose	[41,47,48,49]
		Spring wheat	[50]
	Photoperiod	Pea	[51]
Water stress	Water supply	Rose Arabidopsis	[47,52] [18]
Mineral nutrition	Nitrogen nutrition	Arabidopsis	[53]
		Poplar	[54]
		Rice	[55,56,57,58]
		Rose	[59]
	Phosphorus nutrition	Rice	[60,61]
		Wheat	[62]
	Sulfur nutrition	Arabidopsis	[63]
Temperature	High temperature	Citrus lemon	[64]
CO_2_	High CO_2_	Pea	[65]
